# Single‐Atom Cobalt Species on Curved Hollow Carbon Sphere: Breaking Linear Scaling Relationship Limitations in Electrocatalytic Deactivation of Dilute Antibiotic Pollutants

**DOI:** 10.1002/advs.202522322

**Published:** 2026-01-04

**Authors:** Jiahong Zou, Shenbin Zheng, Chunyang Dan, Huimin Sui, Wenyang Fu, Xiaoshu Lv, Boping Ren, Guangming Jiang, Hong Liu

**Affiliations:** ^1^ Engineering Research Center For Waste Oil Recovery Technology and Equipment Ministry of Education Chongqing Technology and Business University Chongqing China; ^2^ Chongqing Institute of Green and Intelligent Technology Chinese Academy of Sciences Chongqing School University of Chinese Academy of Sciences Chongqing China

**Keywords:** antibiotic pollutant deactivation, antibiotics, hollow porous carbon, linear scaling relationship, single‐atom catalyst

## Abstract

Despite maximal atomic efficiency, single‐atom catalysts (SACs) are constrained by linear scaling relationships in electrocatalytic hydrodechlorination (ECHD), a process effective for antibiotic pollutant deactivation yet challenged by multi‐proton /electron transfer and dilute reactant conditions. Herein, a novel cobalt (Co)‐SACs consisting of single‐atom Co anchored on a nitrogen‐doped hollow porous carbon sphere (Co_1_/N‐HCS) is fabricated, which exhibits a remarkable mass activity of 14.1 g_FLO_ g_Co_^−1^ at −0.30 V toward florfenicol (FLO, a typical antibiotic pollutant), outperforming Co_1_/carbon black, Co nanoparticles/carbon black, and previously reported catalysts. Mechanistic studies reveal that the unique hollow porous architecture of N‐HCS facilitates fluid dynamic enhancement and localized adsorptive enrichment of dilute FLO, while its curved surface boosts proton transfer at Co_1_ through localized electric field enhancement. The dual enhancements enable Co_1_ to break the linear scaling relationship limitations. Field tests for Co_1_/N‐HCS in natural lake water demonstrate excellent environmental stability and matrix interference resistance. Furthermore, it could effectively deactivate dilute FLO (5 µmol L^−1^) while suppressing the emergence of antibiotic resistance gene, highlighting its prospect in antibiotic pollution remediation. This study introduces a paradigm‐shifting support architecture to transcend the SACs performance boundary, while pioneering the application of ECHD for antibiotic pollutant remediation.

## Graphical abstract

A cobalt (Co)‐SACs consisting of single‐atom Co anchored on a nitrogen‐doped hollow porous carbon sphere (Co_1_/N‐HCS) is fabricated for electrocatalytic hydrodechlorination of dilute antibiotics. The hollow porous architecture of N‐HCS concentrates the antibiotics, while its curved surface promotes proton transfer at the Co_1_ site via localized electric field enhancement. The dual enhancements enable Co_1_ to break linear scaling relationship limitations.

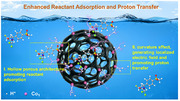

## 1 Introduction

Single‐atom catalysts (SACs) have emerged as a paradigm‐shifting class of materials in heterogeneous catalysis, distinguished by their theoretically 100% atomic utilization efficiency, tunable coordination microenvironments, and unique quantum‐ confined electronic structures [1]. These exceptional characteristics position SACs as transformative solutions for critical applications ranging from renewable energy conversion, biomass upgrading, to environmental remediation (e.g., nitrate reduction, pollutant degradation) [2, 3, 4]. Nevertheless, SACs are fundamentally limited by linear scaling relationships in catalytic performance optimization [5, 6]. This thermodynamic limitation arises from the incapacity of sparse isolated active sites to accommodate multiple intermediates in a multi‐proton/electron transfer process [7, 8]. Recent advances in dual‐site catalysts (DSCs) have effectively improved these limitations through synergistic effects between adjacent active sites (either dual‐single atom pairs or single atom‐nanoparticle configurations), which collectively enhance proton/electron transfer and reduce the energy barrier of rate‐determining steps [9, 10]. For instance, Jia et al. developed a dual‐site catalyst comprising Ru single atoms and nanoparticles (Ru SA/Ru NP) on the same carbon support, which achieved exceptional performance in electrochemical hydrogenation of toluene to methylcyclohexane [11]. They claimed that the Ru NP served to adsorb and activate the toluene, while the Ru‐SA adsorbs protons and transports them to neighboring Ru‐NP for selective hydrogenation. However, the synthesis of dual atomic/NP catalysts typically requires atomic‐level precision in controlling the spatial arrangement of the dual active sites and is often energy‐intensive, thereby hindering their large‐scale application [12].

Integrating a single‐atomic site onto a support material with complementary active sites and additional functions provides a viable strategy to circumvent the limitations imposed by linear scaling relationships. For example, Shen et al. deposited the single‐atom copper (Cu_1_) on a tungsten oxide nanosheet support (Cu_1_/WO_3_), which was highly efficient in the selective conversion of nitrate to ammonia [13]. Integrated experimental and computational studies revealed that the WO_3_ support enhanced proton transfer kinetics through boosting water dissociation, effectively overcoming the proton supply limitation at Cu_1_ sites. WO_3_ was further demonstrated to modulate the electronic structure of Cu_1_ sites through a unique coordination environment, thereby lowering the activation barrier for nitrate reduction. On the other hand, Liu et al. anchored the single‐atom platinum (Pt_1_) on curved onion‐like carbon nanospheres (OLC) for hydrogen evolution [14]. They highlighted the curvature effect of OLC, which induced a strong local electric field around Pt_1_ sites, accelerating proton transfer and promoting hydrogen evolution. Despite the promise of multifunctional supports in developing efficient and cost‐effective DSCs, it remains imperative to explore more robust functional support materials in specific reactions.

Electrocatalytic hydrodechlorination reaction (ECHD) represents a sustainable strategy for deactivation of chlorinated antibiotics (a persistent environmental contaminant that can promote bacterial resistance) via cleavage of carbon‐halogen bonds [15]. Nevertheless, this multi‐proton/electron transfer process, typically occurring in dilute reactant systems, generally suffers from sluggish proton‐transfer kinetics and limited mass diffusion when employing single‐atom catalysts (SACs). To address these challenges, a nitrogen‐doped hollow porous carbon sphere (N‐HCS) was fabricated to host atomically dispersed cobalt species (Co_1_) for ECHD, leveraging its unique structural advantages: (i) the hollow porous architecture might confine and concentrate the dilute antibiotics through adsorptive enrichment [16, 17, 18]; and (ii) the inherent curvature effect facilitated local proton transfer near the Co_1_ sites [19]. The resultant Co_1_/N‐HCS was used for ECHD of florfenicol (FLO, a representative antibiotic contaminant present at trace concentration in wastewater). To assess the promoting effect of N‐HCS, the ECHD performance of Co_1_/N‐HCS toward FLO was tested and compared with that of Co_1_/carbon black (Co_1_/C) and Co NPs/carbon black composites (Co NPs/C). Mechanistic studies combining experimental and theoretical approaches were further conducted to elucidate how the N‐HCS synergized with Co_1_, particularly in the aspects of reactant adsorption, C‐Cl bond activation, mass transport, and proton transfer kinetics. Finally, the practical applicability of Co_1_/N‐HCS was validated by treating FLO‐contaminated lake water, with subsequent analyses of its effects on antibiotic resistance gene (ARG) expression and microbial community succession.

## 2 Results and Discussion

### 2.1 Synthesis and Characterization of the Catalyst

**FIGURE 1:**
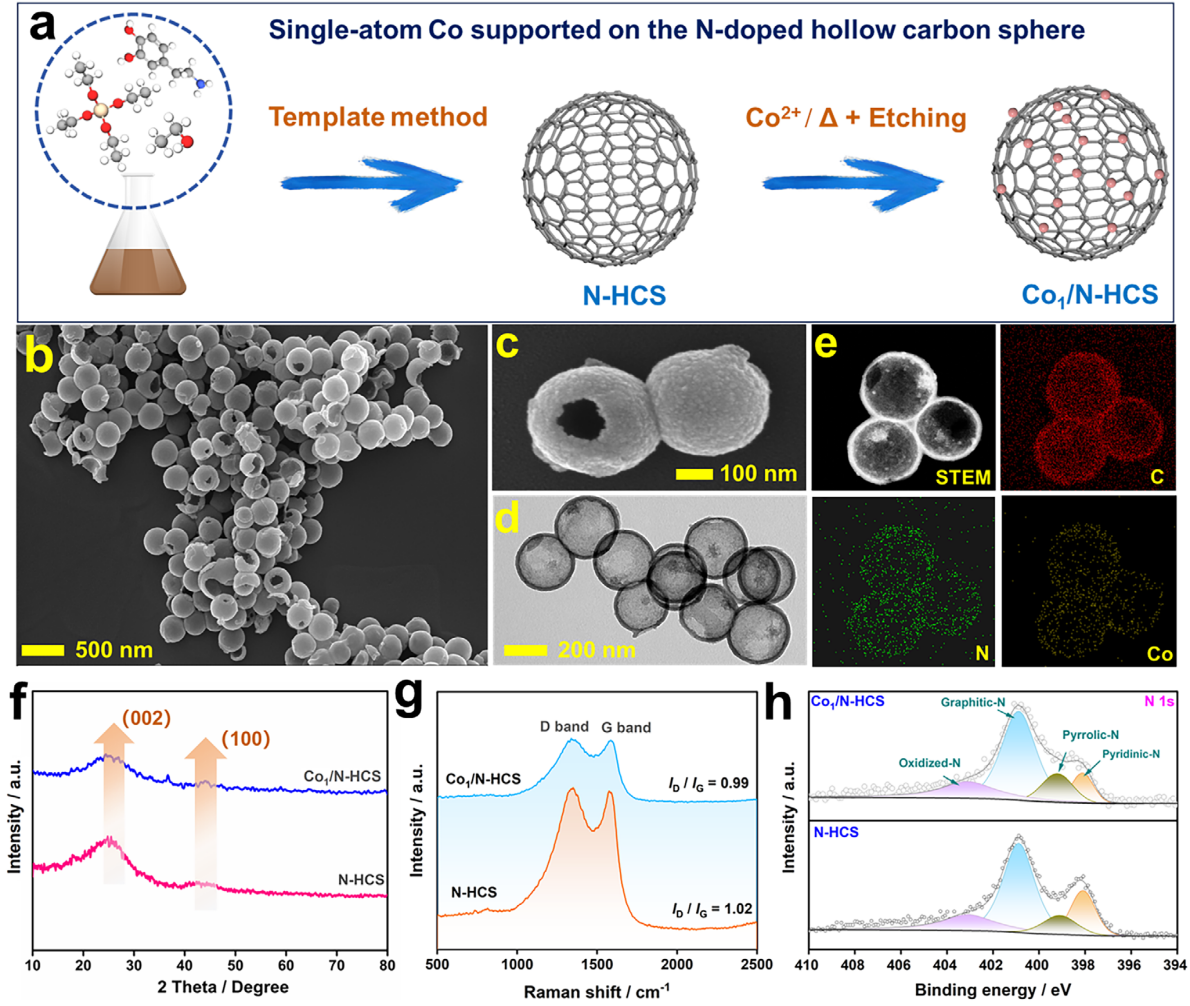
(a) Schematical illustration in the synthetic procedure for Co_1_/N‐HCS; (b),(c) SEM images, (d) TEM image, and (e) Energy‐dispersive X‐ray spectroscopy (EDX) elemental mapping images (C, N, and Co) for Co_1_/N‐HCS; (f) XRD patterns of Co_1_/N‐HCS and N‐HCS; (g) Raman spectra of Co_1_/N‐HCS and N‐HCS; (h) High‐resolution N 1s XPS spectra for Co_1_/N‐HCS and N‐HCS.

Figure 1a depicts the synthetic procedure of Co_1_/N‐HCS with an ultralow Co loading of 0.14%. The fabrication involves the preparation of N‐HCS via a well‐established sacrificial template method, followed by the precise anchoring of an isolated Co atom onto N‐HCS through a carefully optimized adsorption‐ calcination‐etching protocol. The scanning electron microscopy (SEM) images in Figure 1b demonstrate the monodisperse spherical morphology of Co_1_/N‐HCS with a narrow size distribution (diameter: 260–280 nm). The hollow architecture, also clearly visible in both the SEM and transmission electron microscopy (TEM) images (Figure 1c,d), features a thin carbon shell (∼20 nm) with a large pore on it. This unique structure endows Co_1_/N‐HCS with an ultrahigh Brunauer–Emmett–Teller (BET) surface area of 563.9 m^2^ g^−1^ (Figure S2), 2.4 times greater than commercial Ketjen carbon black (232.6 m^2^ g^−1^), facilitating mass transfer for heterogeneous catalysis. The elemental mapping results in Figure 1e reveal the presence of C, N, and Co on Co_1_/N‐HCS. Neither Cu clusters nor particles are resolved owing to their dissolution during the acid etching step. The X‐ray diffraction (XRD) patterns in Figure 1f evidence the graphitic structure for both the N‐HCS and Co_1_/N‐HCS, with no Co‐related phase detected. In the Raman spectra (Figure 1g), two broad peaks at ∼1360 and 1590 cm^−1^, marked as D and G band, respectively, can be observed. Their intensity ratio (*I*_D_/*I*_G_) was 1.02 and 0.99 for N‐HCS and Co_1_/N‐HCS, respectively, indicating fewer defects and a lower degree of disorder within them even after the inclusion of Co_1_. Their intensity ratio (*I*_D_/*I*_G_) was calculated to be 1.02, indicating fewer defects and a lower degree of disorder within N‐HCS, owing to sufficient polymerization process under the high‐temperature annealing. The high‐resolution N 1s X‐ray photoelectron spectroscopy (XPS) spectra (Figure 1h) reveal four distinct nitrogen configurations in N‐HCS: oxidized N (403.1 eV), graphitic N (400.9 eV), pyrrolic N (399.2 eV), and pyridinic N (398.1 eV). Remarkably, upon Co_1_ incorporation, while the relative contents of the other three N species remain essentially unchanged, the pyridinic N component shows a pronounced reduction, suggesting its preferential coordination with Co single atoms during the anchoring process. This selective decrease provides evidence for the formation of Co‐N_x_ moieties, consistent with previous reports on single‐atom catalysts [20].

**FIGURE 2:**
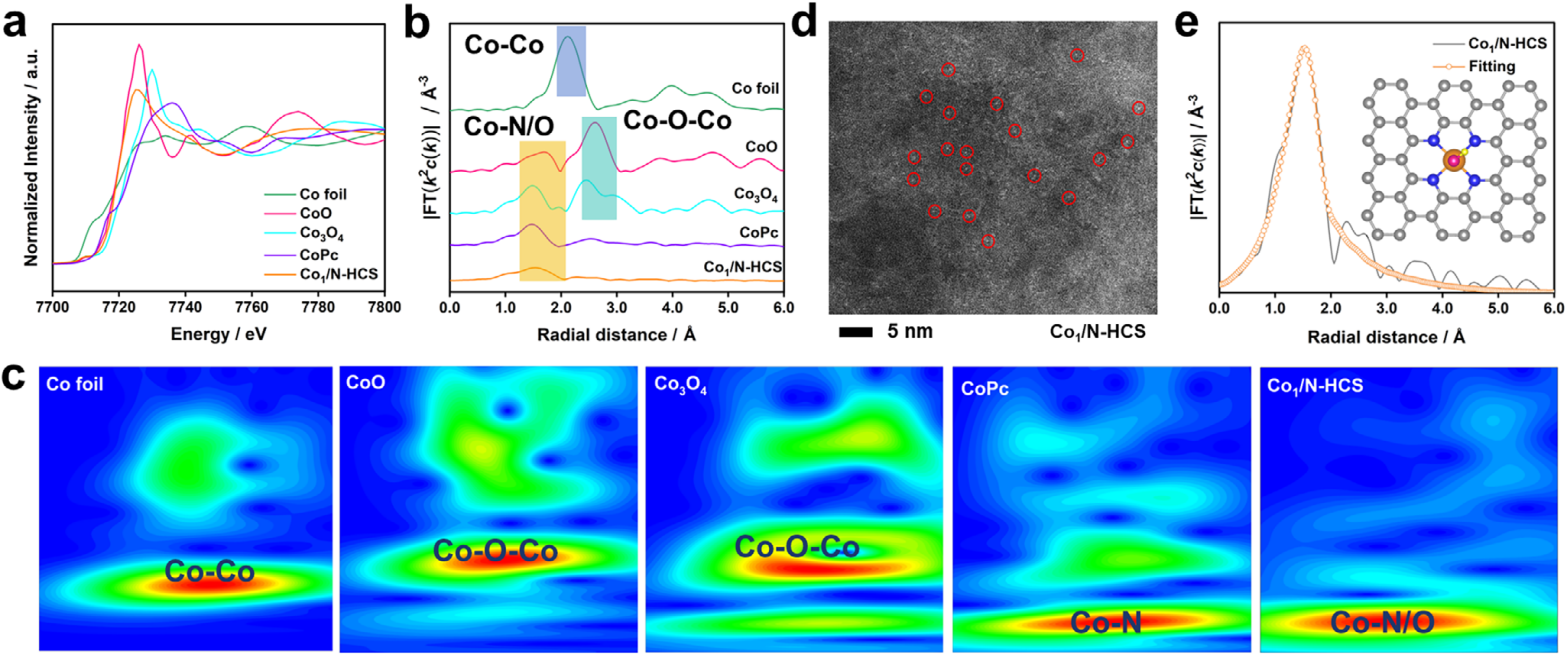
(a) Normalized Co K‐edge XANES spectra, (b) k^3^‐weight FT‐EXAFS spectra and (c) WT‐EXAFS spectra for Co_1_/N‐HCS and reference samples, respectively; (d) HAADF‐STEM image of the Co_1_/N‐HCS; (e) Fitting results of the FT‐EXAFS spectra for Co_1_/N‐HCS.

To determine the local coordination state of Co_1_ in Co_1_/N‐HCS, X‐ray absorption spectroscopy (XAS) analyses were further performed [21, 22]. Figure 2a shows the X‐ray absorption near‐edge structure (XANES) spectra of Co_1_/N‐HCS and the reference Co foil, CoO, Co_3_O_4_, and the cobalt phthalocyanine (CoPc) with a unique Co‐N_4_ moiety. The *K*‐edge absorption of Co_1_/N‐HCS lies between those of CoPc, CoO, and Co_3_O_4_ but closer to that of CoO, implying that the chemical valence of Co_1_ approaches +2. Figure 2b shows that the Fourier‐transformed extended X‐ray absorption fine structure (FT‐EXAFS) spectrum of Co_1_/N‐HCS resembles that of CoPc, featuring a prominent scattering peak at approximately 1.5 Å. This peak is commonly assigned to the first coordination shell of the Co‐O/N bond, which is also observed for CoO, Co_3_O_4_, and CoPc. The scattering peaks at ∼2.2, 2.8, and 3.0 Å, corresponding to the Co‐Co bond in the Co foil, CoO, and Co_3_O_4_, are not resolved, suggesting the absence of any Co‐based clusters. The wavelet transform (WT) results in Figure 2c confirm the presence of only an isolated Co atom in Co_1_/N‐HCS. Further verification of the atomic dispersion of Co was provided by High‐angle annular dark‐field scanning transmission electron microscopy (HAADF‐STEM), which revealed distinct bright dots in Co_1_/N‐HCS corresponding to isolated Co single atoms in Figure 2d. Furthermore, the contour plot of Co_1_/N‐HCS closely resembles that of CoPc, demonstrating that the Co_1_ is primarily bonded to N atoms. The fitting results from FT‐EXAFS features in Figure 2e and Table S1 show that the first shell of the central Co atom displays a coordination number of 5.0 with four N toms (with fitted Co‐N bond length of 2.08 Å) and one hydroxyl (with fitted Co‐O bond length of 1.95 Å). All the above experimental and simulation results reveal that the Co_1_ with a unique N_4_‐Co_1_‐OH coordination configuration has been incorporated into N‐HCS.

### 2.2 ECHD Performance Evaluation

**FIGURE 3:**
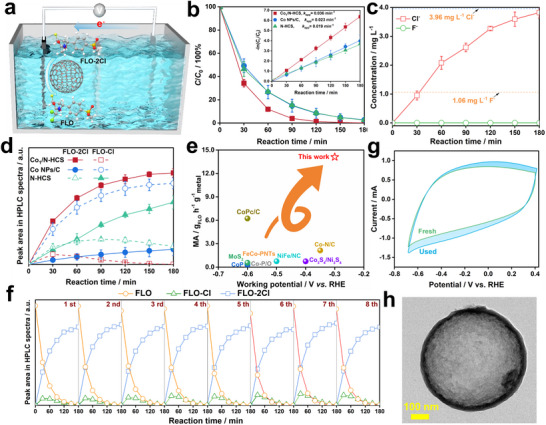
(a) Schematic illustration of the ECHD reaction system; (b) Plots of *C*/*C*_0_ with reaction time during ECHD of FLO on N‐HCS, Co_1_/N‐HCS, and Co NPs/C; (c) Variation in the peak areas of FLO‐2Cl and FLO‐Cl and (d) the yields of Cl^−^ and F^−^ against reaction time during ECHD; (e) Comparison in the mass activity between Co_1_/N‐HCS and the catalysts reported in literature [8, 23, 24, 25, 26, 27, 28, 29, 30]; (f) Durability test for Co_1_/N‐HCS; (g) CV curves of the fresh and used Co_1_/N‐HCS; (h) TEM image of the Co_1_/N‐HCS after cycled tests.

The Co_1_/N‐HCS catalyst was tested for ECHD of FLO at a dilute concentration of 55.8 µmol L^−1^ under a constant potential of −0.30 V (Figure 3a). Its performance was compared to that of N‐HCS and Co NPs/C. The *C/C*_0_ evolution profiles for FLO as a function of reaction time in Figure 3b show that the FLO is almost eliminated on all three catalysts within 180 min. The inset plots ‐ln(*C/C*_0_) vs reaction time, and the displayed linear relationship demonstrates that the ECHD on the three catalysts follow the pseudo‐first‐order reaction model. The Co_1_/N‐HCS displays the highest apparent kinetic constant (0.037 min^−1^), followed by Co NPs/C (0.024 min^−1^) and N‐HCS (0.019 min^−1^), indicating that the ECHD on Co_1_/N‐HCS is less limited by the mass diffusion of FLO. To probe into the ECHD pathway for FLO, the intermediate organics and ions (Cl^−^ and F^−^) were quantified with High‐performance liquid chromatography (HPLC) and ionic chromatography (IC) techniques, respectively. The reaction time‐dependent HPLC patterns in Figure S3 and IC spectra in Figure 3c demonstrate that the C‐Cl bonds, rather than the C‐F bond, are cleaved on the three catalysts, owing to the significantly higher strength of the C‐F bond. Figure 3d summarizes the reaction time‐dependent variation in the peak areas for FLO‐Cl (one C‐Cl bond cleaved) and FLO‐2Cl (two C‐Cl bond cleaved) on the HPLC spectra. The peak area of FLO‐Cl exhibits a volcano‐like variation trend, while that of FLO‐2Cl increases gradually with reaction time. Intriguingly, in the Co_1_/N‐HCS system, the FLO‐Cl disappears, and the FLO‐2Cl reaches its maximum yield at the end of the reaction, suggesting its superior ECHD performance compared to single N‐HCS and Co NPs/C. Based on the amount of FLO converted to FLO‐2Cl, the mass activity of Co_1_/N‐HCS is calculated to be 14.1 g_FLO_ g_Co_^−1^, which makes it exceptionally stand out among the catalysts reported in literature (Figure 3e). Durability is another critical criterion for describing the ECHD performance. As observed in Figure 3f, the profiles of the amount variations for FLO, FLO‐Cl, and FLO‐2Cl as a function of reaction time across the eight cycles of batch tests show minimal differences, demonstrating that Co_1_/N‐HCS exhibited durable ECHD activity. The Co leaching into the solution in the first cycle was 1.87 µg L^−1^, much below the threshold concentration (Class I: 10 µg L^−1^) required by the Environmental quality standards for surface water (GB 3838‐2002). Figure 3g shows minimal differences in the shape and size of the CVs of Co_1_/N‐HCS before and after cycling tests. The TEM image of the Co_1_/N‐HCS after cycling tests in Figure 3h shows that the Co_1_/N‐HCS maintains the original morphology and size, and more importantly, no Co‐based particles are resolved. All these demonstrate that the Co_1_/N‐HCS is chemically stable in the mild reaction environment.

To comprehensively evaluate the practical applicability of Co_1_/N‐HCS, systematic ECHD performance assessments were conducted under varying working potentials, electrolyte concentrations, FLO concentrations, and in the presence of ionic impurities and dissolved organic matter (DOM) that is generally contained in wastewater. Figure S4a,b shows that the ECHD efficiency improved significantly when the applied potential was shifted negatively from −0.10 to −0.30 V, attributable to the enhanced reducing power of electrons at more cathodic potentials. However, a further shift to −0.40 V slowed down the ECHD, possibly owing to the more prevailed side hydrogen evolution reaction (HER) under this reaction condition. The vigorous gas evolution from HER created bubbles that physically blocked reactant transport from the bulk solution to the catalyst surface, thereby limiting mass transfer of FLO and protons to the active sites [30]. Figure S4c,d shows that increasing the electrolyte (Na_2_SO_4_) concentration from 10 to 50 mm significantly boosted the ECHD of FLO. Similar phenomena have been widely reported in electrochemical systems, attributed to enhanced solution conductivity and optimized electrical double‐layer structure at the electrode–electrolyte interface [31]. Figure S4e,f reveal comparable FLO conversion rates across the tested concentration range (5.6–55.8 µmol L^−1^), demonstrating that Co_1_/N‐HCS maintains high efficiency over a broad pollutant concentration window. Figure S4g,h demonstrate that common wastewater impurities, including Cl^−^ (5 mm), NO_3_^−^ (1 mm), CO_3_^2−^ (5 mm) at environmentally relevant concentrations, exhibited negligible interference on ECHD kinetics over Co_1_/N‐HCS. To evaluate the impacts of DOMs, humic acid (HA) was employed as a representative model compound. Figure S5a shows that the removal rate of FLO decreased as the HA concentration increased from 10 to 100 mg L^−1^. Nevertheless, complete conversion of FLO was achieved within 180 min under all conditions. Figure S5b indicates that the ECHD kinetics of the two C‐Cl bonds were minimally affected by the presence of 10 mg L^−1^ FLO. However, at higher concentrations (50 and 100 mg L^−1^), an increased yield of the intermediate FLO‐Cl was observed. These findings demonstrate that our Co_1_/N‐HCS exhibits robust fouling resistance against organic compounds, albeit within a certain concentration limit. Collectively, all these results illustrate that the Co_1_/N‐HCS possesses a wide operational potential window in complex aqueous matrices.

### 2.3 Reaction Mechanism

**FIGURE 4:**
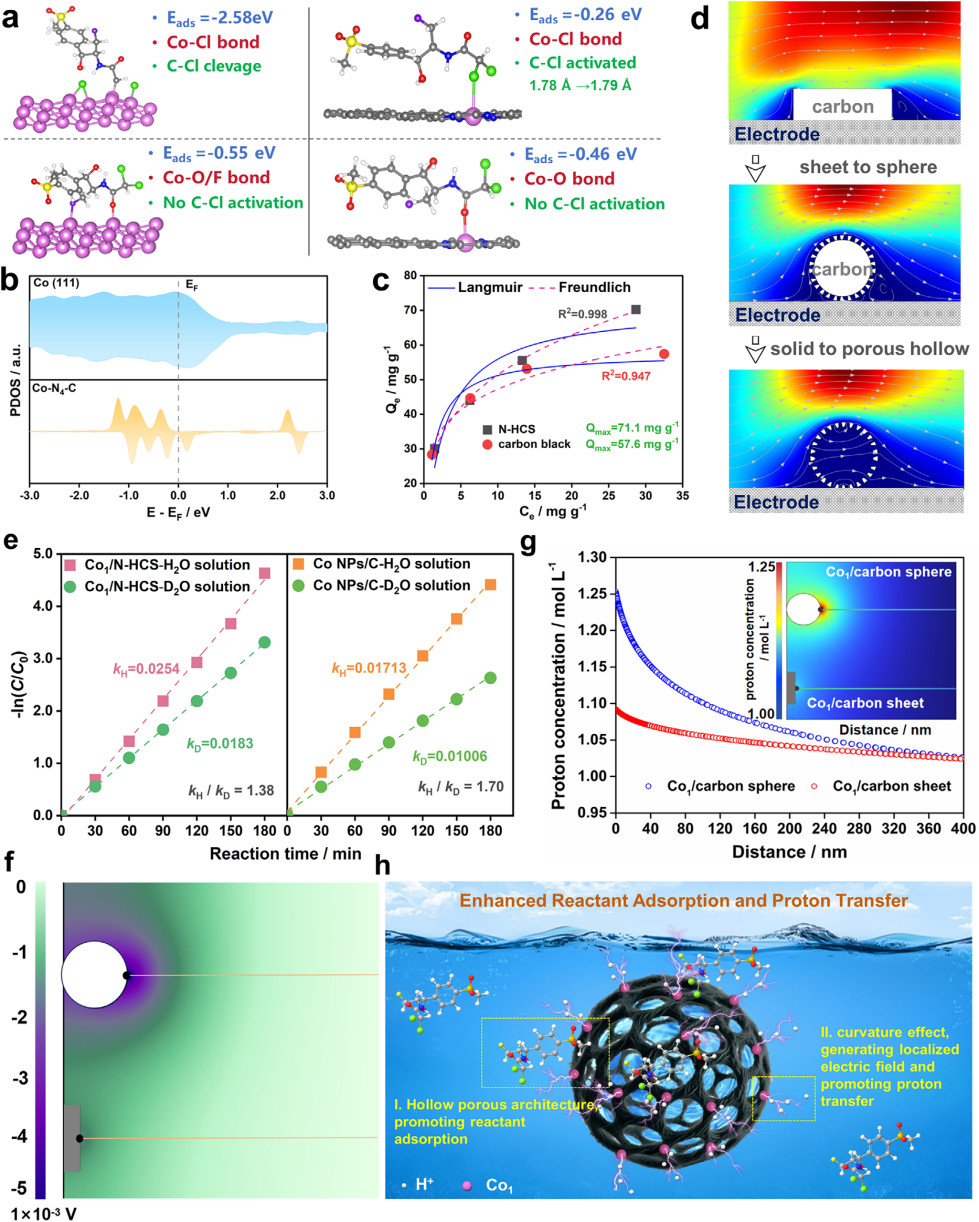
(a) Configurations and interaction behaviors of FLO adsorption on Co(111) and graphene layer with Co‐N_4_ moiety (Co‐N_4_‐C). (b) partial densities of states of the Co for Co (111) and Co‐N_4_‐C, respectively; (c) Adsorption isotherms recorded for FLO on Co_1_/N‐HCS and Co NPs/C; (d) Micron‐scale flow behaviors around carbon sheet, solid carbon sphere, and hollow porous carbon sphere, respectively. (e) KIE plots of ECHD rate constant ratio (KIE = k_H2O_/k_D2O_) on Co_1_/N‐HCS and Co NPs/C; (f),(g) Simulated longitudinal distribution of the localized electric field and H^+^ concentration from electrode surface to bulk solution for Co/carbon sphere and Co/carbon sheet, respectively (The pH in bulk solution was set to 6.0). (h) Schematical illustration of the critical role of N‐HCS in promoting the ECHD performance of Co_1_ site.

To elucidate the role of N‐HCS, the intrinsic reactivities of the Co_1_ site and Co NPs were comparatively analyzed. Initially, the electron transfer pathway from the Co_1_ site to FLO in ECHD, encompassing both direct electron transfer (DET) and hydrogen radical (H^*^)‐mediated indirect manner, was investigated. The CV patterns in Figure S6a show that after adding FLO, a characteristic reduction current peak, associated with the hydrolysis of C‐Cl bonds, appears at approximately −0.30 V. Additionally, Figure S6b shows that there are minimal differences in ECHD kinetics in the absence and presence of 50 mm TBA (an efficient H^*^ quencher). Both results indicate that ECHD on the Co‐based catalysts proceeds via the DET pathway. Upon this electron transfer manner, the reactivity difference between Co_1_ and Co NPs can be described through their distinct interactions with FLO. As evidenced in Figure 4a, the Co (111) surface exhibits enhanced FLO adsorption and more efficient C‐Cl bond activation relative to the Co_1_ site (modeled by Co‐N_4_ moiety embedded in graphene support, abbreviated as Co_1_‐N_4_‐C), with one adsorption configuration even showing spontaneous bond cleavage. The present larger interaction intensity on Co (111) can be attributed to two key factors: First, the extended Co (111) surface offers multiple geometrically compatible adsorption sites with strong binding affinity for the bulky FLO molecule. Second, electronic structure analyses (Figure 4b) reveal that while the Co_1_ site exhibits localized discrete *d‐*orbital states characteristic of atomic‐scale coordination, metallic Co demonstrates delocalized continuum states with significant density of states near the Fermi level. This metallic electronic structure facilitates enhanced d‐p orbital hybridization through improved energy matching between Co and the FLO molecule. Both experimental and simulation results demonstrate that the Co NPs with extended surface outperform Co_1_ in FLO molecule conversion. This conclusion is to some extent supported by the results that the Co NPs/C outperforms Co_1_/C in terms of the FLO‐2Cl yield under identical conditions (Figure S7). In this context, the superior performance of Co_1_/N‐HCS should originate from the N‐HCS support.

As a hollow porous spherical support, the N‐HCS was expected to improve reactant accessibility to catalytic sites by combining localized adsorptive enrichment of target molecules and fluid dynamic enhancement within its hollow porous architecture. Figure 4c displays the Langmuir and the Freundlich isotherm curves for the adsorption of FLO on N‐HCS and carbon black, respectively. The fitting results and correlation coefficients (R^2^) of the respective isotherm models indicate that the adsorption of FLO on both carbon materials follows the Freundlich isotherm model more closely, suggesting a heterogeneous multilayer adsorption mechanism. The maximum adsorption capacity (Q_max_) of N‐HCS was determined to be 71.1 mg g^−1^, which is 1.25 times higher than that of carbon black. The enhanced adsorption capacity promotes the enrichment of dilute species near the reactive sites, contributing to an enhanced ECHD efficiency. To elucidate the influence of N‐HCS on microscale fluid dynamics, computational fluid dynamics (CFD) simulations were performed to systematically compare flow patterns around N‐HCS with those of carbon sheets and solid carbon spheres (Details in Supporting Information) [32]. As illustrated in Figure 4d, the turbulent flow regime (highlighted in red) near the three configurations exhibits minimal difference, which can be attributed to the negligible differences in catalyst size. Nevertheless, a thicker laminar flow region is observed near the catalyst surface of the two spherical samples compared to the carbon sheet, suggesting that the spherical configuration enhances solution retention on the catalyst surface. Moreover, the N‐HCS architecture provides a unique confinement effect, enabling controlled infiltration of the electrolyte solution into the hollow cavity at a significantly reduced flow rate. The reduced flow velocity, induced by both the spherical shape and hollow porous structure, collectively prolongs the catalyst‐FLO interaction time, thereby sustaining highly efficient electron‐proton transfer processes.

Given the substantial consumption of protons, the ECHD reaction is often kinetically limited by proton transfer, a process dominated by water dissociation at the catalytic interface [33]. Herein, kinetic isotope effect (KIE) studies were first conducted to elucidate the dependence of ECHD efficiency on proton transfer dynamics for both the Co_1_/N‐HCS and Co NPs/C [34]. The effect is quantificationally described as the ratio of pseudo‐first‐order rate constants for FLO removal in H_2_O versus D_2_O system (*k*_H_/*k*_D_). A *k*_H_/*k*_D_ ratio substantially greater than 1.0 indicates pronounced kinetic isotope effects in the ECHD process, revealing strong sensitivity to proton transfer dynamics and implicating proton‐coupled electron transfer as the rate‐determining step [35]. Figure 4e reveals that while both Co_1_/N‐HCS and Co NPs/C systems exhibit kinetic isotope effects (*k*_H_/*k*_D_ >1.0), the significantly lower ratio observed for Co_1_/N‐HCS (1.38 vs 1.70 for Co NPs/C) demonstrates that the N‐HCS support contributes to proton transfer around the Co_1_ site. The enhanced proton transfer kinetics were corroborated by both linear sweep voltammetry (lsv) and electrochemical impedance spectroscopy (EIS) studies. As shown in Figure S8a, Co_1_/N‐HCS exhibits consistently higher reductive current densities than Co_1_/C over the potential range of 0.2 to −1.0 V, indicating superior hydrogen evolution activity attributable to facilitated proton transfer. Furthermore, the EIS spectra in Figure S8b reveal similar charge transfer resistance (R_ct_) for both catalysts but a significantly larger diffusion resistance for Co_1_/C, as evidenced by the steeper low‐frequency slope [36]. These findings collectively demonstrate that the N‐HCS structure efficiently promotes proton mass transport. To investigate how the N‐HCS promotes proton transfer, finite element method (FEM) simulations were performed using COMSOL Multiphysics, building upon recent advancements in interfacial field engineering [37]. A metallic Co site anchored on a carbon sphere (Co_1_/carbon sphere) was used to model Co_1_/N‐HCS, while a Co site on a flat carbon sheet (Co_1_/carbon sheet) represented Co_1_/C. Figure 4f demonstrates that electron charging (cathodic polarization) to the two configurations induces substantial electron accumulation at the metallic Co sites, generating an intense localized electric field in their vicinity. Notably, the Co_1_/carbon sphere interface demonstrates a substantially higher electrical potential than the Co_1_/carbon sheet system. This enhancement can be attributed to the well‐documented curvature effect arising from the curved surface of the carbon sphere [14, 38]. This effect has been demonstrated to simultaneously enable reactant enrichment and activation around the catalyst, significantly promoting various electrocatalytic processes including hydrolysis and hydrogenation reactions [39, 40]. To probe the influence of localized electric fields on proton transfer dynamics, a coupled electrostatic‐diffusion module was employed to investigate the proton distributions at the Co_1_/carbon sphere and Co_1_/carbon sheet surfaces, respectively (See details in Supporting Information). Figure 4g reveals significantly enhanced proton accumulation around the Co_1_/carbon sphere (1.25 µmol L^−1^) compared to the Co_1_/carbon sheet (1.09 µmol L^−1^). These findings clearly demonstrate that the N‐HCS support facilitates proton transfer kinetics near the Co_1_ site through curvature‐induced effects.

Collectively, these findings establish that the N‐HCS support is pivotal for boosting ECHD performance of Co_1_ sites. As illustrated in Figure 4h, its contribution arises from two synergistic mechanisms: (i) the hierarchical hollow porous architecture facilitates efficient diffusion and localized enrichment of FLO near the Co_1_ active centers; (ii) the curved surface geometry induces a localized intense electric field at the Co_1_ site that accelerates the proton transfer. Together, these two effects enable Co_1_ sites to overcome the intrinsic limitations imposed by the linear scaling relationship, achieving exceptional efficiency in ECHD of the dilute FLO contaminants.

### 2.4 Environmental Implications

**FIGURE 5:**
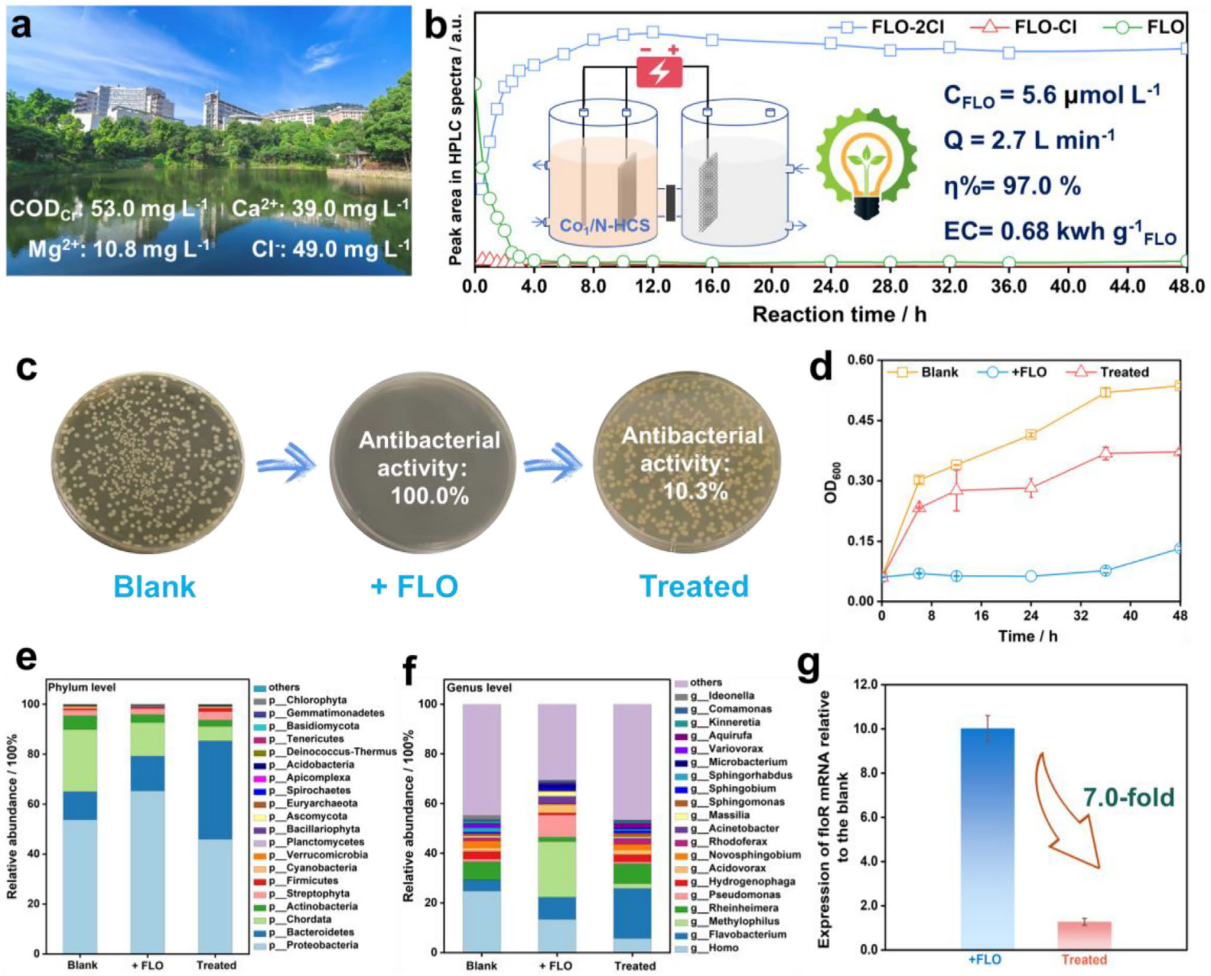
(a) The quality of water sampled from the Cuihu Lake located in Chongqing, China; (b) ECHD treatment for the contaminated lake water sample in a continuous‐flow reaction cell; (c) Photographs of the agar plates with *E. coli* and the culture from pure water, the contaminated water and the water treated by ECHD;(d) OD600 of the *E. coli*‐involved culture during the 48 h incubation process; Microbial community abundance on (e) phylum level and (f) genus level; (h) Expression of floR mRNA relative to the blank.

The Co_1_/N‐HCS catalyst was employed for FLO remediation in natural water sampled from Chongqing Cuihu Lake, demonstrating its potential for real‐world environmental applications. Figure 5a shows that the water sample contains dissolved organic matter (COD_Cr_ = 53 mg L^−1^), Ca^2+^ (39 mg L^−1^), Mg^2+^ (10.8 mg L^−1^), and Cl^−^ (49.0 mg L^−1^). Prior to experiments, some FLO (5.6 µmol L^−1^) and electrolytes (20 mm Na_2_SO_4_) were added to the sample. The results in Figure 5b show that the system achieves 97% FLO removal under continuous flow operation with a mild energy consumption of 0.68 kwh g^−1^, while generating negligible amounts of FLO‐Cl in effluent [8]. Notably, the Co_1_/N‐HCS maintained stable performance throughout the 48‐h continuous reaction, demonstrating excellent operational durability when exposed to the natural water. The ECHD process was expected to reduce the antibiotic activity of the FLO‐contaminated water. Antibacterial assessment using *E. coli* (ATCC 25922, a model Gram‐negative bacterium) reveals that ECHD treatment reduces the antibacterial activity of the contaminated water from 100% (complete growth inhibition) to 10.3% (Figure 5c) [41]. This 89.7% reduction confirms efficient deactivation of the antibiotics, attributable to hydrolysis of critical C‐Cl bonds in the FLO molecule [25]. OD600 measurements further confirm these findings, with *E. coli* growth in treated water approaching uncontaminated control levels and significantly exceeding untreated samples throughout the 48‐h culture period (Figure 5d).

Impacts of ECHD treatment on microbial community succession and floR gene abundance in water sample was investigated [42]. As shown in Figure 5e, FLO exposure caused reductions in most phyla's relative abundance (especially the actinobacteria and chordata) versus the control. In contrast, Proteobacteria (increasing from 53.7% to 65.0%) and Bacteroidetes (from 11.3% to 13.9%) show moderate enrichment, suggesting FLO‐resistant phenotypes. In the treated samples, while most bacterial phyla exhibit decreased abundance compared to the control and +FLO groups, Bacteroidetes showed significant enrichment, potentially attributable to its metabolic capacity for FLO‐2Cl degradation. At the genomic level, Figure 5f demonstrates selective enrichment of FLO‐resistant genera, with Methylophilus showing the highest increase (290.1‐fold), followed by Pseudomonas (7.4‐fold), Acinetobacter (38.8‐fold), and Flavobacterium (1.0‐fold), suggesting these taxa possess inherent or acquired FLO tolerance mechanisms. After treatment, most genera exhibit reduced abundance, with the notable exception of Flavobacterium, which demonstrates significant enrichment (1.2‐fold increase). This selective advantage likely stems from Flavobacterium's genomic capacity for FLO‐2Cl biodegradation [27]. Meanwhile, other taxa rebound to near‐baseline levels, confirming that the ECHD system achieves targeted FLO deactivation without disrupting ecological equilibrium. Further analyses of floR gene abundance reveal a significant reduction in the ECHD‐treated sample relative to the FLO‐contaminated control (Figure 5g). This decline (7.0‐fold decrease) aligns with the cleavage of C‐Cl bonds in FLO. The attenuation of floR suggests that the ECHD can mitigate the risk of antibiotic resistance gene propagation by disrupting the structural integrity of FLO required for resistance selection pressure.

## 3 Conclusion

In summary, we developed a novel Co_1_/N‐HCS catalyst with exceptional efficiency and durability in ECHD of FLO. The catalyst delivered an outstanding mass activity of 14.1 g_FLO_ g_Co_^−1^ at −0.30 V toward 55.8 µmol L^−1^ FLO, which outperformed Co_1_/C and Co NPs/C counterparts, and previously reported catalysts in literature. The exceptional catalytic efficiency could be attributed to the distinctive hollow porous structure and curved surface of the N‐HCS. The 3D hollow porous architecture promoted efficient FLO diffusion and localized concentration enrichment, while the curved surface geometry enhanced proton transfer by inducing a strong localized electric field at the Co_1_ sites. These synergistic effects collectively enabled the Co_1_ to break the inherent linear scaling relationship limitations in ECHD. Field tests in lake water confirmed Co_1_/N‐HCS's exceptional environmental stability and matrix interference resistance. Furthermore, the ECHD process effectively deactivated FLO while suppressing ARG emergence, demonstrating strong potential for antibiotic pollution remediation. This study establishes a novel multifunctional support strategy to break activity limitations of SACs, coupled with the transformative application of ECHD technology for environmental antibiotic remediation.

## Conflicts of Interest

The authors declare no conflicts of interest.

## Supporting information




**Supporting File**: advs73683‐sup‐0001‐SuppMat.docx.

## Data Availability

The data that support the findings of this study are available in the supplementary material of this article.
