# Supplementary material for: Single‐Atom Cobalt Species on Curved Hollow Carbon Sphere: Breaking Linear Scaling Relationship Limitations in Electrocatalytic Deactivation of Dilute Antibiotic Pollutants
Source: Adv Sci (Weinh). 2026 Jan 4;13(16):e22322. doi: 10.1002/advs.202522322 (PMC13042813; doi:10.1002/advs.202522322)
Supplement: Supplementary file 1 — Supporting File: advs73683‐sup‐0001‐SuppMat.docx. [file ADVS-13-e22322-s001.docx]

**Single-atom Cobalt Species on Curved Hollow Carbon Sphere: Breaking Linear Scaling Relationship Limitations in Electrocatalytic Deactivation of Dilute Antibiotic Pollutants**

*Jiahong Zou^a^, Shenbin Zheng^a^, Chunyang Dan^a^, Huimin Sui^a^, Wenyang Fu^a^, Xiaoshu Lv^a^, Boping Ren^a^, Guangming Jiang^a*^, Hong Liu^b,*^*

*^a^Engineering Research Center for Waste Oil Recovery Technology and Equipment, Ministry of Education, Chongqing Technology and Business University, Chongqing 400067, China*

*^b^Chongqing Institute of Green and Intelligent Technology, Chinese Academy of Sciences,*

*Chongqing School, University of Chinese Academy of Sciences, Chongqing 400714, China*

^*^Corresponding author:

**E-mail:** [jiangguangming@zju.edu.cn;](mailto:jiangguangming@zju.edu.cn;) [liuhong@cigit.ac.cn](mailto:liuhong@cigit.ac.cn)

**S1. Materials and Methods**

**1.1. Materials**. Analytical sodium sulfate (Na_2_SO_4_), ethanol, tert-Butanol, hydrochloric acid (HCl), orthophosphoric acid (H_3_PO_4_) and chromatographical-grade methanol were obtained from Sinopharm Group Chemical Reagent Co., Ltd., China. Analytical Florfenicol (FLO, C_12_H_14_Cl_2_FNO_4_S) and 3-hydroxytyramine hydrochloride (C_8_H_12_ClNO_2_) were supplied with Shanghai Aladdin Reagent Co., Ltd., China. Deuterium oxide (D_2_O) and Nafion solution (5 wt%) were provided by Sigma-Aldrich. Tetraethoxysilane (TEOS, C_8_H_20_O_4_Si) and ammonia (NH_3_^.^H_2_O) were purchased from Shanghai Titan Scientific Co., Ltd., China. Carbon paper (Toray 060) obtained from Cabot Co., Ltd., China. Ketjen carbon black (Vulcan XC 72R) was provided by Cabot.

**1.2. Synthesis of the Catalyst and Working Electrode**

**Synthesis of N-HCS**. Typically, 1.0 mL of TEOS, 1.0 mL of NH_3_**^.^**H_2_O and 2.0 mmol of 3-hydroxytyramine hydrochloride aqueous solution (0.258 mol L^-1^) were dissolved in a mixed solvent of water (80 mL) and ethanol (24 mL) under vigorous stirring. After a reaction of 24.0 h, yellow powders were collected through filtration, washed with H_2_O and dried at 60 ^o^C. These powders were further calcined in a tube furnace at 800 ^o^C for 2 h under nitrogen atmosphere with a ramping rate of 5 ^o^C min^-1^. Upon cooling to room temperature, the resulting grey powder was dispersed in 10 mL of 10 wt% HF solution via ultrasonication. Following a visible color transition from grey to black, the product was separated by filtration, thoroughly washed with deionized water and dried under vacuum, yielding the N-HCS product.

**Synthesis of Co_1_/N-HCS,** **Co NPs/C and Co_1_/C**. For Co_1_/N-HCS, 60.0 mg of N-HCS was uniformly dispersed in 80 mL of ethanol via ultrasonication, yielding an ink-like solution. 20 mL of an ethanolic Co(NO_3_)_2_**^.^**6H_2_O solution (15.6 mg) was added dropwise to the ink under sonication. The mixture was stirred vigorously at 70 ^o^C until the solvent was evaporated. The resultant solid phase was calcined at 400 ^o^C for 1.0 h in a tube furnace under nitrogen atmosphere with a ramping rate of 5 ^o^C min^-1^. After cooling to room temperature, the solid phase was collected and then subjected to 10 mL HCl solution (1.0 mol L^-1^) to remove Co particles (acid etching step). After stirring for 30 min, the product was collected by filtration, washed with H_2_O and dried to obtain Co_1_/N-HCS. The Co NPs/C was synthesized using an identical preparation method, with carbon black substituted for N-HCS and omitting the acid etching step. The Co_1_/carbon black was prepared by treating the Co NPs/C in 10 mL HCl solution (1.0 mol L^-1^). The Co_1_ loading in Co NPs/C and Co_1_/carbon black was 4.99% and 1.02%, respectively. The TEM images of Co NPs/C and Co_1_/C were presented in **Fig. S1**.

**Preparation of Working Electrode.** The working electrode was prepared following a published work.^[20]^ Typically, 13.5 mg of catalyst was dispersed in a mixed solvent of ethanol (3.0 mL), and Nafion (30.0 µL) under intense sonication. The resultant ink was dripped onto the surface of a carbon paper substrate (accessible area: 2 cm × 2 cm), and the solvent in the ink was gradually evaporated by irradiating the electrode with an infrared heating lamp. A uniform catalyst film was then formed on the carbon paper, forming the working electrode.

**1.3. Performance Evaluation of the Catalysts**. The ECHD tests were conducted in an H-type cell, which was separated to a cathode chamber and an anode chamber by a proton-exchange membrane. A conventional three-electrode system was employed, comprising the catalyst-modified working electrode (cathode), an Ag/AgCl (3 M KCl) reference electrode, and a platinum foil counter electrode, with potential control relying on an electrochemical workstation. The working potentials present below were further calibrated against the reversible hydrogen electrode (RHE): E_RHE_ = E_Ag/AgCl_ + 0.20 V + 0.0592 × pH. Prior to tests, both chambers were charged with 40 mL of nitrogen-saturated Na_2_SO_4_ solution, and the cathode chamber was additionally charged with specified amount of FLO. At predetermined time intervals, aliquots (typically 0.5-1.0 mL) of the reaction solution were collected for quantitative analyses of FLO and reaction products. The removal efficiency (*η*%) of FLO was calculated as:

*η*% = (*C*_0, FLO_ - *C*_t, FLO_) / *C*_0,FLO_ × 100% (1)

where *C*_0, FLO_ and *C_t_*_, FLO_ (mg L^-1^) refer to the FLO concentration at the time of 0 and *t* min. The mass activity of the catalyst (MA, g_FLO_ h^-1^ g_Co_^-1^) was expressed by:

MA = (*C*_0, FLO_ - *C*_180, FLO_) × (0.04 L) / (m_Co_ × 180 min) (2)

where m_Co_ denotes the Co mass loading on electrode (g) .

The energy consumption (EC) (k_Wh_ g_FLO_^-1^) for removing every gram of FLO in the continuous-flow reaction cell was calculated as

EC = E_cell_ × I/[(*C*_in,FLO_ - *C*_out,FLO_) × Q] (3)

where the E_cell_, I and Q refer to the cell voltage (V), current (A) and flow velocity (L h^-1^), respectively. *C*_in,FLO_ and *C*_out,FLO_ represent the concentration of FLO (g L^-1^) in the influent and effluent, respectively.

**1.4. Characterization.** The transmission electron microscopy (TEM), scanning transmission electron microscopy (STEM) and energy dispersive X-ray (EDX) elemental mapping images were obtained on JEM-F200, Japan. Scanning electron microscopy (SEM) images were obtained on TESCAN MIR 3, Czech Republic. Aberration-corrected high-angle annular dark-field scanning transmission electron microscopy (HAADF-STEM) images were obtained on JEOL ARM 200F, Japan. X-ray diffraction (XRD) patterns were recorded on an X-ray diffractometer (Model D/max RA, Rigaku Co., Japan) under Cu Kα radiation. The catalyst composition and the amount of Co leaching in solution were determined by inductively coupled plasma-atomic emission spectroscopy (ICP2060t, Tianrui Co., China). The electronic state of nitrogen atoms in the catalyst was characterized by X-ray photoelectron spectroscopy (XPS, Thermo ESCALAB 250, USA) equipped with Al Kα X-rays (hν=1486.6 eV) operated at 150 W. The X-ray absorption fine structure (XAFS) analyses were performed in a fluorescence mode at the beamline BL14W1 of the Shanghai Synchrotron Radiation Facility in China. Raman spectroscopy was conducted on Horiba LabRAM HR Evolution (HORIBA Scientific Co., Japan). The BET specific surface areas of samples were measured with an Pm2-1533-A nitrogen adsorption apparatus (Beishide Instrument Technology Co.,China). All electrochemical tests, including cyclic voltammetry (CV), linear sweep voltammetry (LSV), electrochemical impedance spectroscopy (EIS) and ECHD tests, were performed on a electrochemical workstation (CHI660E, Chenhua Co., China). The concentration of FLO and intermediate products were determined by the high-performance liquid chromatography (HPLC, SHIMADZU 2010-AT, Japan) equipped with a UV detector (225 nm) and an ODS-SP column (150 × 4.6 nm). The mobile phase contained the water, methanol and orthophosphoric acid (V:V:V=1:1:0.0008) at a flow rate of 0.5 mL min^-1^. The chloride and fluoride ions (Cl^-^ and F^-^) were quantified by an ion chromatography (Thermo Fisher Scientific ICo., USA). The culture optical density (OD600) was measured by a multimode microplate reader (BioTek, Cytation5, USA).

**S2. Experimental section**

**S2.1. Density Functional Theory (DFT) Calculations**

All calculations were carried out in the framework of Density Functional Theory (DFT) using the Vienna Ab initio Simulation Package.^[1-3]^ The generalised gradient approximation (GGA) of the Perdew-Burke-Ernzerhof (PBE) function was used to describe the exchange-correlation energy.^[4]^ The projector augmented wave (PAW) method was used to describe the interactions between electrons and ions,^[5]^ and a plane wave basic set with cutoff energy of 400 eV was set to expand electron states. 3×3×1, 2×3×1 Monkhorst-Pack k points grids for the Co (111) surfaces and Co-N_4_ embedded in graphene were employed, respectively. The lattice parameters and ionic positions of all crystals were fully relaxed, and the convergence criteria for the total energy of all relaxed atoms and the final force were 10^-5^ eV and 0.03 eV/Å, respectively. The adsorption energy (∆E) of adsorbed species can be defined by

∆E = E_tot_ - (E_sub_ + E_ads_) (4)

Where E_tot_, E_sub_ and E_ads_ describe the energies of the surface with adsorbate, the clean surface and the isolated absorbate, respectively.

**S2.2. Finite Element Analysis (FEA) Simulation**

FEA was conducted utilizing the computational fluid dynamics in COMSOL Multiphysics 5.6. The mass transfer phenomenon within the system was numerically simulated by solving the incompressibl Navier-Stokes equations. The carbon black, solid carbon sphere, and N-HCS were modeled as a rectangle (82 nm×28 nm), a solid circle (120 nm diameter), and a porous circular ring (120 nm outer diameter, 20 nm shell thickness), respectively. The fluid was assigned a density of 1,000 kg m^-3^, a viscosity of 1.01×10^-^³Pa^.^s and a velocity of 1.1 m s^-1^.

**S2.3. Finite-element Method (FEM) Simulation**

FEM was performed in COMSOL Multiphysics 5.6. The Static Electricity and Transport of Diluted Species modules were coupled to model the interfacial potential distribution and ion transport. The Co_1_/N-HCS and Co_1_/C catalysts were modeled as single metallic Co sites anchored on a hollow carbon sphere (denoted as Co_1_/carbon sphere) and a flat carbon sheet (denoted as Co_1_/carbon sheet), respectively.During the simulation, both catalyst surfaces were charged with a uniform surface charge density of -1×10⁻⁴ C/m². The diffusion coefficient was set to 5.85×10⁻⁹ m² s^-1^ for H⁺.


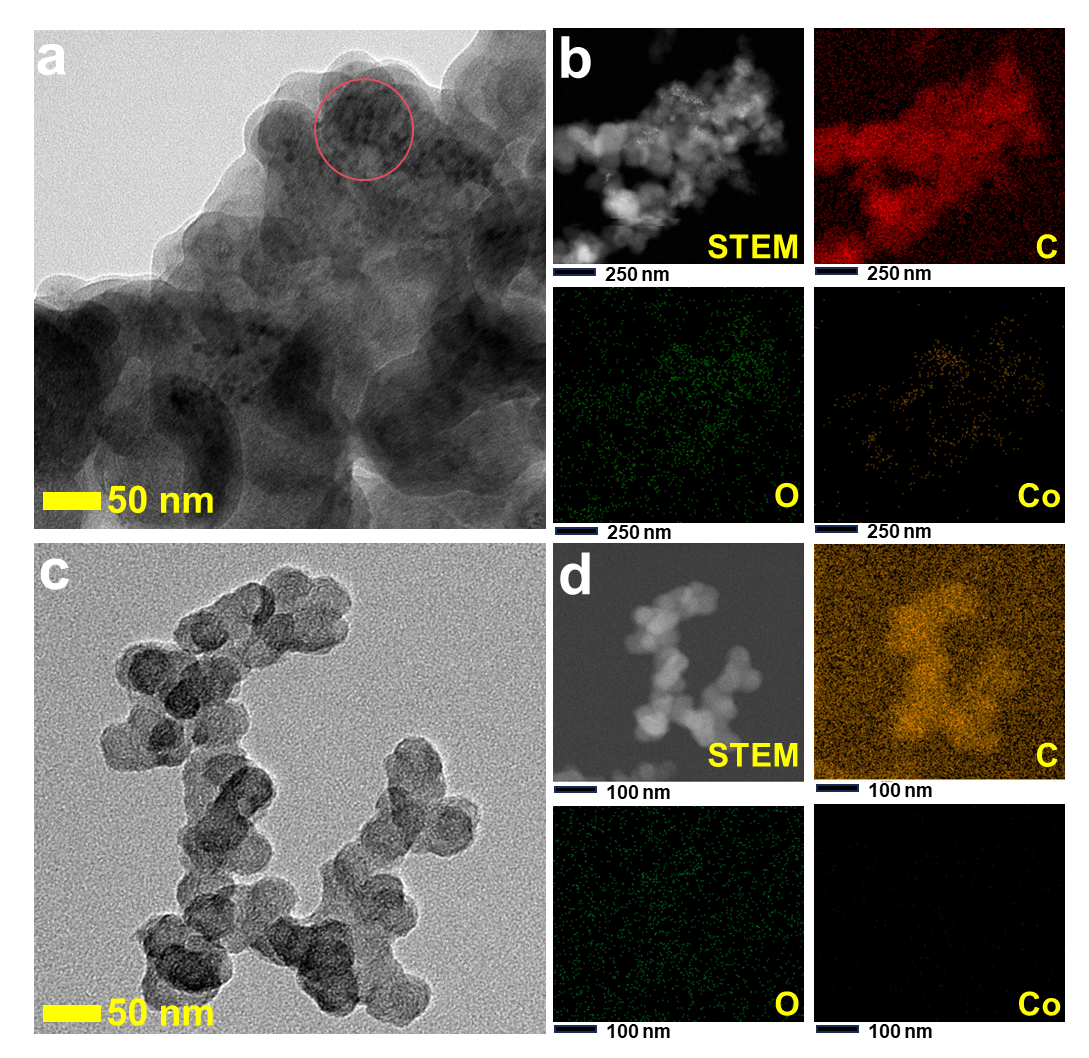


**Figure S1.** (a) TEM of Co NPs/C; (b) EDX elemental mapping of C, O and Co of the Co NPs/C; (c) TEM of Co_1_/C; (d) EDX elemental mapping of C, O and Co of the Co_1_/C.

**
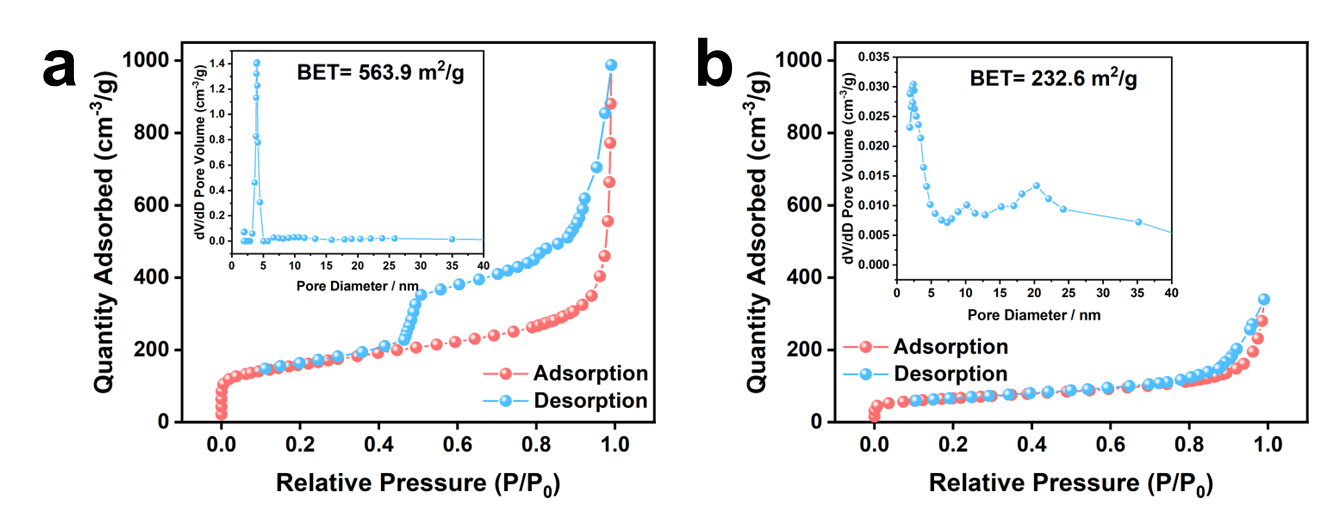
**

**Figure S2.** Nitrogen adsorption-desorption isotherms and the pore size distribution for (a) N-HCS and (b) carbon black.


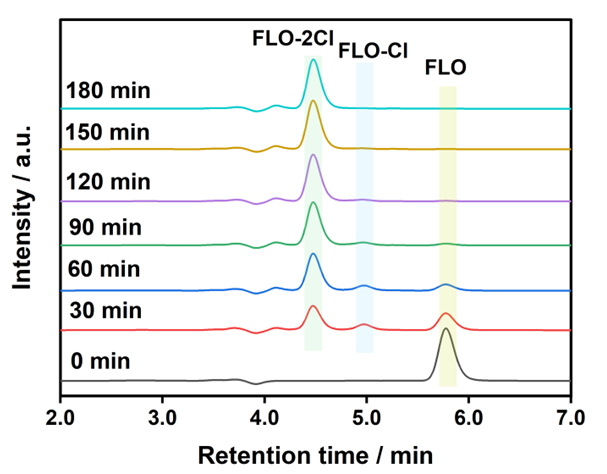


**Figure S3.** The reaction time dependent HPLC spectra for Co_1_/N-HCS at different reaction time.


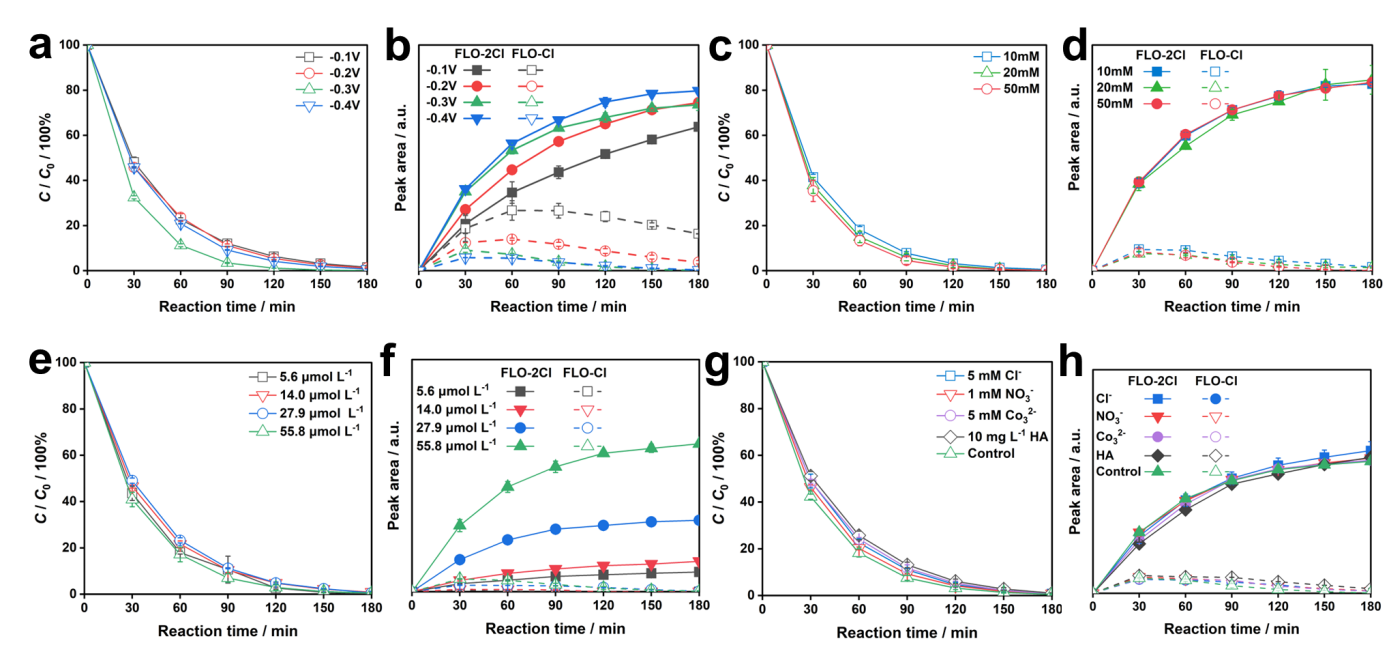


**Figure S4.** Impacts of (a-b) working potential, (c-d) Na_2_SO_4_ concentration, (e-f) feeding FLO concentration and (g-h) Coexsting impurity ions and dissolved organics (represented by humic acid, HA) on the kinetics and product distribution during ECHD on Co_1_/N-HCS.


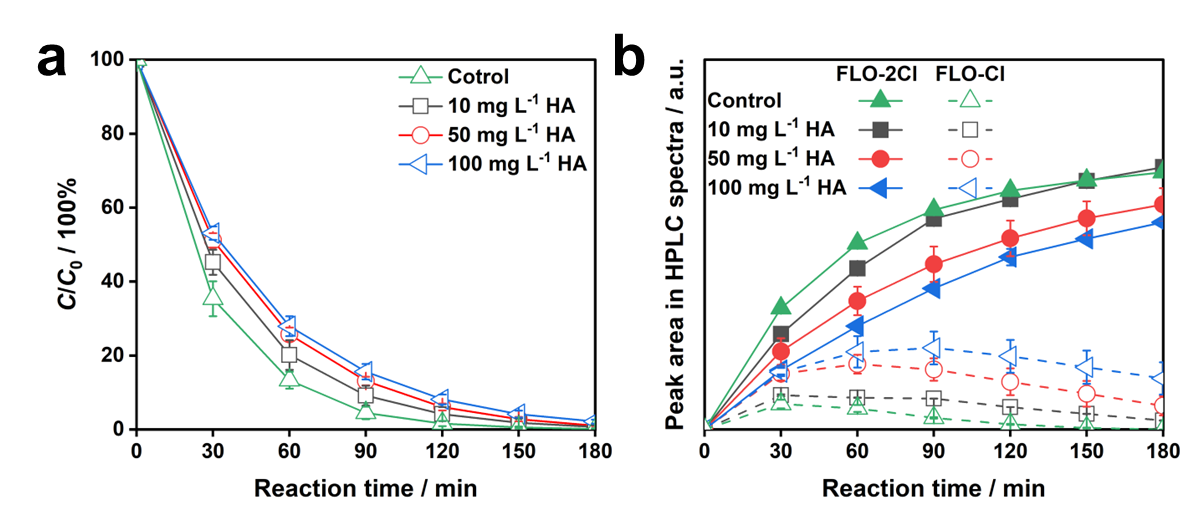


**Figure S5.** (a) humic acid concentration on the kinetics and (b) product distribution during ECHD on Co_1_/N-HCS.


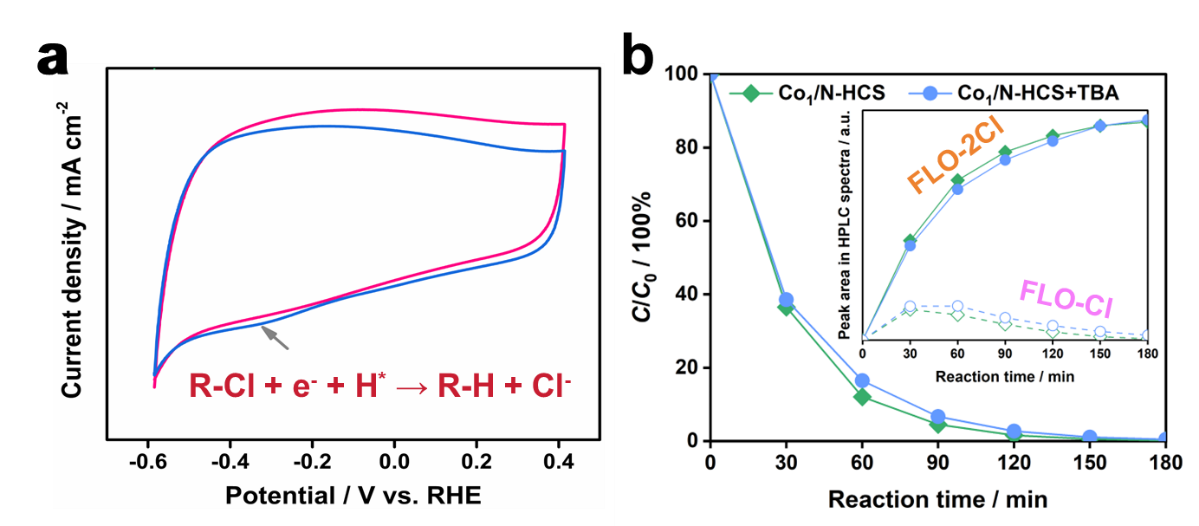


**Figure S6.** (a) CV curves of Co_1_/N-HCS with and without FLO; (b) Plotting of *C*/*C*_0_ against reaction time during ECHD of FLO in the absence and presence of TBA (50 mM).


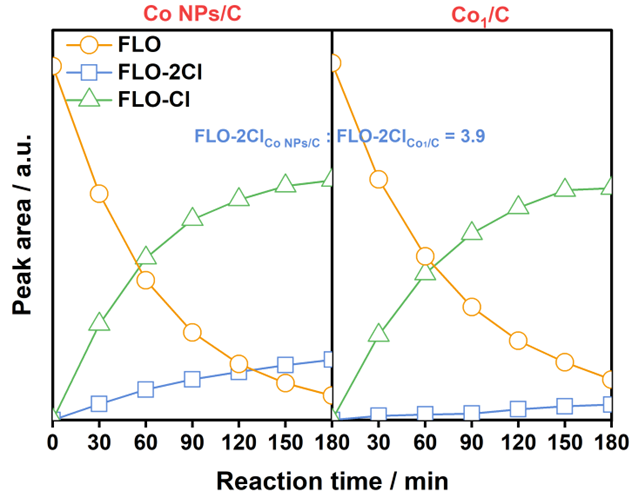


**Figure S7.** Variation in the peak areas of FLO, FLO-2Cl and FLO-Cl against reaction time during ECHD on Co NPs/C and Co_1_/C.

**
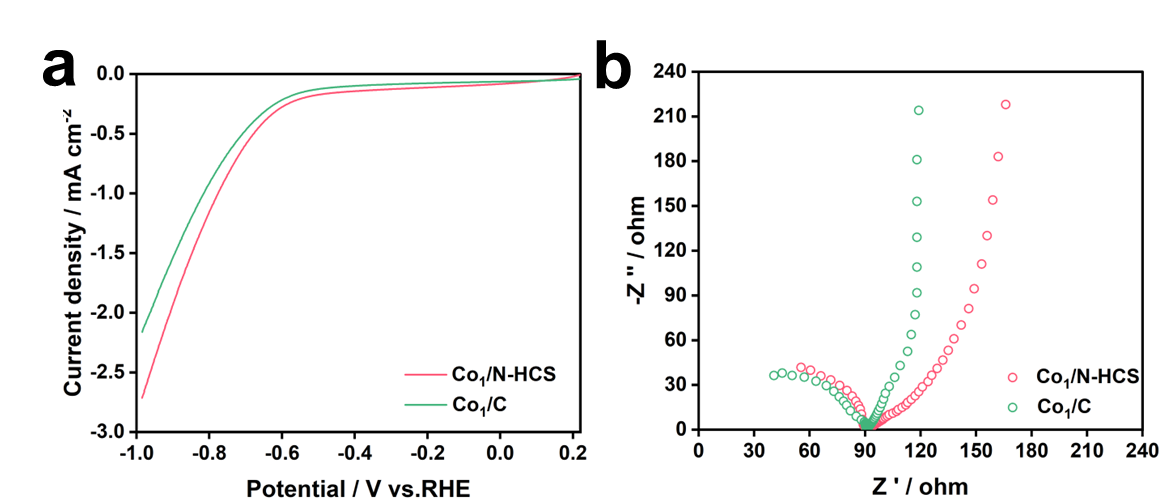
**

**Figure S8.** (a) LSV curves for Co_1_/N-HCS and Co_1_/C; (b) Nyquist plots of capacitive currents vs scan rates of Co_1_/N-HCS and Co_1_/C.

**Table S1** EXAFS fitting parameters at the Co K–edge for various samples

| **Sample** | **Path** | ***^a^CN*** | **^b^R（Å）** | **^c^σ^2^（🞨10^-3^ Å^2^）** | | ***^d^ΔE*_0_（ev）** | **R-factor** |
| --- | --- | --- | --- | --- | --- | --- | --- |
| Co foil | Co-Co | 12 | 2.49(0.002) | 6.18(0.23) | 7.63(0.32) | | 0.003 |
| CoPc | Co-N | 4 | 1.92(0.01) | 2.45(1.42) | 3.95(2.21) | | 0.021 |
|  | Co-C | 8 | 2.94(0.02) | 1.01(2.79) |  |  |  |
| CoO | Co-O | 6 | 2.10(0.02) | 3.32(1.77) | 6.83(0.67) | | 0.016 |
|  | Co-Co | 12 | 3.01(0.01) | 6.02(0.52) |  |  |  |
| Co_3_O_4_ | Co-O | 4 | 1.93(0.03) | 3.92(7.04) | 6.68(0.36) | | 0.011 |
|  | Co-Co | 12 | 2.88(0.002) | 6.06(0.27) |  |  |  |
| Sample-Co | Co-N | 3.86(0.41) | 2.08(0.04) | 8.05(11.03) | 2.70(2.05) | | 0.017 |
|  | Co-O | 1.15(0.14) | 1.95(0.03) |  |  |  |  |

*^a^CN*, coordination number; *^b^R*, distance between absorber and backscatter atoms; *^c^σ*^2^, Debye-Waller factor to account for both thermal and structural disorders; *^d^ΔE*_0_, inner potential correction; *R* factor indicates the goodness of the fit. S_0_^2^ was fixed to 0.74, according to the experimental EXAFS fit of Co foil by fixing CN as the known crystallographic value. A reasonable range of EXAFS fitting parameters: 0.600 < *Ѕ*_0_^2^ < 1.000; *CN >* 0; *σ*^2^ > 0 Å^2^; |Δ*E*_0_| < 15 eV; *R* factor < 0.02.

**Table S2** Comparison in activity of various catalysts.

| **Catalysts** | **C_FLO_/**  **mg L^-1^** | **Potential**  **(vs. RHE)** | **Mass activity**  **(μg_FLO_ h^-1^ g^-1^)** | **Stability** | **Sel.%** | **Ref** |
| --- | --- | --- | --- | --- | --- | --- |
| CoPc/C | 20.0 | -0.6 V | 6.23 | 48 hours | 100% | [1] |
| Co-N/C | 10.0 | 2.5 mA cm^-2^ | 2.14 | stable in 10 cycled batch tests | 98.7% | [2] |
| Co_3_S_4_/Ni_3_S_4_ | 20.0 | -0.4 V | 0.74 | stable in 5 cycled batch tests | 77% | [3] |
| MoS_2_ | 20.0 | -0.6 V | 0.56 | stable in 5 cycled batch tests | 83% | [4] |
| NiFe@NC | 20.0 | -0.5 V | 0.75 | 7 hours | 100% | [5] |
| FeCo-PNTs | 20.0 | -0.6 V | 0.41 | stable in 8 cycled batch tests | 100% | [6] |
| C-CoP/Ti | 20.0 | -0.6 V | 0.31 | stable in 5 cycled batch tests | 100% | [7] |
| Co-P/O | 20.0 | -0.6 V | 0.29 | stable in 25 cycled batch tests | 91% | [8] |
| **Co_1_/N-HCS** | **20.0** | **-0.3 V** | **14.1** | **48 hours** | **100%** | **This work** |

**Reference:**

1. Liu, Y., Tang, X., Shen, F., Zou, J., Yin, Y., Jiao, Z., Lv, X., Fu, W., Zou, Y., Jiang, G., Liu, H., Cobalt single-site molecular Catalyst-mediated electrochemical Hydrodechlorination for detoxification of halogenated Antibiotics: Performance, reaction pathway and mechanism. Chemical Engineering Journal 499(2024), 156276.
2. G. Song, H.Z. Wu, J.A. Jing, X.Y. Zhang, X.C. Wang, S.S. Li, M.H. Zhou, Insights into Electrochemical Dehalogenation by Non-Noble Metal Single-Atom Cobalt with High Efficiency and Low Energy Consumption, *Environ. Sci. Technol.* 57(38) (2023) 14482-14492.

[3] S. Wu, L. Wan, C.H. Luo, Y.X. Hu, S.Y. Zhang, S.A. Baig, X.H. Xu, Electrocatalytic dechlorination of Florfenicol using crystalline Co3S4/Ni3S4 nanowires arrayed on nickel foam via cathodic reduction, *J. Electroanal. Chem.* 948 (2023) 9.

[4] J. Yang, S.-F. Jiang, W.-F. Hu, H. Jiang, Highly efficient electrochemical dechlorination of florfenicol by an ultrathin molybdenum disulfide cathode, *Chem. Eng. J.* 427 (2022) 131600.

[5] D. Zhang, Y. Tang, H. Liu, Z. Wang, X. Liu, H. Tang, H. Zhang, D. Wang, Y. Long, C. Liu, Electrocatalytic Deep Dehalogenation and Mineralization of Florfenicol: Synergy of Atomic Hydrogen Reduction and Hydroxyl Radical Oxidation over Bifunctional Cathode Catalyst, *Environ. Sci. Technol.* 57(48) (2023) 20315-20325.

[6] H.L. Liu, Y.C. Ding, H.F. Tang, Y. Du, D.Y. Zhang, Y.H. Tang, C.B. Liu, Electrocatalytic deep dehalogenation of florfenicol using Fe-doped CoP nanotubes array for blocking resistance gene expression and microbial inhibition during biochemical treatment, *Water Res.* 201 (2021) 9.

[7] H.L. Liu, J.L. Han, J.L. Yuan, C.B. Liu, D. Wang, T. Liu, M.J. Liu, J.M. Luo, A.J. Wang, J.C. Crittenden, Deep Dehalogenation of Florfenicol Using Crystalline CoP Nanosheet Arrays on a Ti Plate via Direct Cathodic Reduction and Atomic H, *Environ. Sci. Technol.* 53(20) (2019) 11932-11940.

[8] T. Liu, J. Luo, X. Meng, L. Yang, B. Liang, M. Liu, C. Liu, A. Wang, X. Liu, Y. Pei, J. Yuan, J. Crittenden, Electrocatalytic dechlorination of halogenated antibiotics via synergistic effect of chlorine-cobalt bond and atomic H^*^, *J. Hazard. Mater.* 358 (2018) 294-301.
